# The Efficacy and Safety of the Combined Therapy of Sodium-Glucose Co-Transporter-2 Inhibitors and Angiotensin Receptor-Neprilysin Inhibitor in Patients With Heart Failure With Reduced Ejection Fraction: A Meta-Analysis of the EMPEROR-Reduced and DAPA-HF Sub-Analysis

**DOI:** 10.3389/fcvm.2022.882089

**Published:** 2022-05-18

**Authors:** Yanxia Lin, Huanrui Zhang, Shijie Zhao, Ling Chen, Jinyang Li, Xiaoou Wang, Wen Tian

**Affiliations:** Department of Geriatric Cardiology, The First Affiliated Hospital of China Medical University, Shenyang, China

**Keywords:** sodium-glucose co-transporter-2 inhibitors, angiotensin receptor-neprilysin inhibitor, combined therapy, heart failure, meta-analysis

## Abstract

**Background:**

Both sodium-glucose co-transporter-2 (SGLT-2) inhibitors and angiotensin receptor-neprilysin inhibitor (ARNI) were recommended to treat heart failure with reduced ejection fraction (HFrEF). However, no trial was conducted to assess the efficacy and safety of the combined therapy of SGLT-2 inhibitors and ARNI in patients with HFrEF.

**Methods:**

We performed a meta-analysis of the prespecified subgroups from DAPA-HF and EMPEROR-Reduced trials. The primary endpoint was the composite risk of cardiovascular death or hospitalization for heart failure. The risk of cardiovascular death, all-cause death, a composite of serious adverse renal outcomes, and volume depletion were also estimated.

**Results:**

The risk of the composite of cardiovascular death or hospitalization for heart failure was reduced in combined therapy of SGLT-2 inhibitors and ARNI, compared with ARNI monotherapy (RR.68, 95% CI.53 to.85, *P* = 0.001). When compared with SGLT-2 inhibitors monotherapy, the events of cardiovascular death (RR.64, 95% CI.46 to 0.87, *P* = 0.005) and all-cause death (RR.72, 95% CI.55 to.94, *P* = 0.01) were significantly less in combined therapy, accompanied by elevated incidence of volume depletion (RR 1.55, 95% CI 1.22 to 1.96, *P* = 0.0003).

**Conclusion:**

Combined therapy has additional benefits over monotherapy in patients with HFrEF, however, it is accompanied by a possibly higher risk of volume depletion.

## Introduction

Extensive research has shown that sodium-glucose co-transporter-2 (SGLT-2) inhibitors, such as dapagliflozin, empagliflozin, and angiotensin receptor-neprilysin inhibitor (ARNI)-sacubitril/valsartan, have a beneficial effect on reducing the risks of cardiovascular death and hospitalization for heart failure (1–6). Therefore, the 2021 ESC guidelines for heart failure recommended ARNI as a replacement for angiotensin-converting enzyme inhibitor (ACEI) and the SGLT-2 inhibitors, dapagliflozin, and empagliflozin, for patients with HFrEF to reduce the risk of worsening HF and CV death (7).

In the PARADIGM-HF trial, the primary endpoint (death from cardiovascular causes or hospitalization for heart failure) was significantly lower in the sacubitril/valsartan group than in the enalapril group (21.8% vs. 26.5%) (1). Compared to placebo, the risk of cardiovascular death and hospitalization among patients with heart failure and a reduced ejection fraction (HFrEF) in the SGLT-2 inhibitors group was decreased by 26% and 25% in the DAPA-HF and EMPEROR-Reduced trials, respectively (5, 6). Despite the respective benefit of ARNI or SGLT-2 inhibitors, however, no solid evidence of the superiority of the combination of the two medications was demonstrated to any one of them alone for patients with HFrEF.

In a previous cross-trial analysis, the beneficial effects of comprehensive prognosis-improving pharmacological therapy (β blocker, mineralocorticoid receptor antagonists, ARNI, and SGLT-2 inhibitors) are demonstrated and the combined therapy was proposed as a new standard therapeutic approach for HFrEF (8). However, the aggregate effects of the combined therapy might be overestimated because of the methods of indirect comparisons and the assumption of additive benefits from the different medications. A recent meta-analysis based on EMPEROR-Reduced and DAPA-HF trials demonstrated the effects of SGLT-2 inhibitors vs. placebo in patients who were taking sacubitril/valsartan or not, but the effects of the combined therapy were not compared with SGLT-2 inhibitors or ARNI alone. In addition, the endpoint events of safety were seldomly analyzed in this study (9).

**Figure 1:**
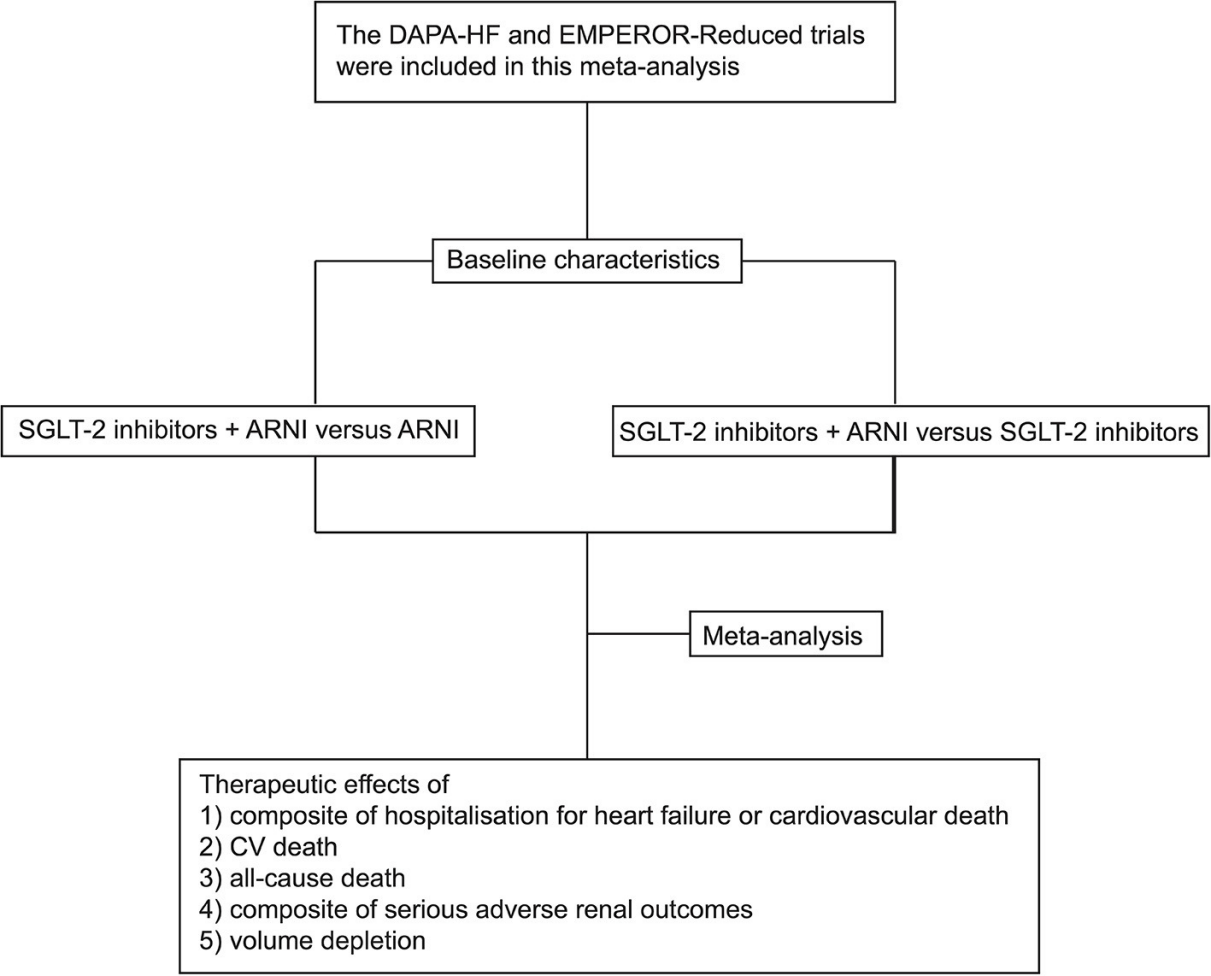
The flow chart of the study.

Considering that the two classes of agents have completely different modes of action, it is uncertain that a combination of the two would benefit more than one drug alone, although concurrent trials reveal that treatment with both SGLT-2 inhibitors and sacubitril/valsartan is well-tolerated (8). In the prespecified neprilysin inhibition sub-group analysis from DAPA-HF and EMPEROR-Reduced trials, the effects of empagliflozin to decrease the cardiovascular death and hospitalization for heart failure are similar in patients who are receiving sacubitril/valsartan or not, however, the benefit of dapagliflozin to reduce the primary endpoint events was not statistically different from placebo for the patients taking sacubitril/valsartan previously (10, 11). The discordant evidence from trials with nearly the same design, together with some controversial points of view on the combined therapy of ARNI and SGLT-2 inhibitors (10–13), warrant further investigation and meta-analysis. Therefore, the main aim of this study is to evaluate the clinical efficacy and safety of combined therapy of SGLT-2 inhibitors and sacubitril/valsartan in patients with HFrEF. The flow chart of the study is illustrated in Figure 1.

## Methods

In this study, we performed a meta-analysis using data acquired from the DAPA-HF and EMPEROR-Reduced trials to evaluate the efficacy and safety of the combination therapy of SGLT-2 inhibitors and ARNI compared with any one of them alone in the patients with HFrEF. Since DAPA-HF and EMPEROR-Reduced are the only randomized controlled trials to evaluate the combined treatment of SGLT-2 inhibitors and ARNI in patients with HFrEF, no additional literature search was conducted. Both the DAPA-HF and EMPEROR-Reduced trials were randomized, double-blind studies that included patients aged over 18 years with low ejection fraction (EF ≤ 40%), elevated NT-proBNP, New York Heart Association (NYHA) class II, III, or IV symptoms (5, 6).

In the DAPA-HF trial, 4,744 patients met the inclusion criteria and were recruited into the study to compare dapagliflozin 10 mg with a placebo. A total of 508 patients (10.7%) received sacubitril/valsartan at baseline. In the EMPEROR-Reduced trial, 3,730 patients of HFrEF were enrolled and 727 patients (19.5%) were already being treated with sacubitril/valsartan at baseline. All the patients treated with sacubitril/valsartan in both trials were prespecified to be randomized into SGLT-2 inhibitors or placebo group. The median duration of follow-up was 18.2 and 16 months in the two trials, respectively.

In order to depict the baseline characteristics of the patients taking ARNI or not, we summarize the baseline data from the two trials and analyze the differences between the two kinds of patients. Based on the published dataset we then categorized these patients into a group of combined treatment with SGLT-2 inhibitors and ARNI, monotherapy group of SGLT-2 inhibitors or ARNI, and placebo groups (10). Thereafter, the efficacy and safety events are compared between the patients with combined therapy and monotherapy groups. Because of the overwhelming superiority of SGLT-2 inhibitors and ARNI to placebo, the comparison with placebo was not performed. Two independent reviewers abstracted data from the included studies, using an assessment form that was designed in advance. Disagreements between the two reviewers were resolved through consultation.

The primary endpoint was the risk of the composite of hospitalization for heart failure or cardiovascular death. We also estimated the risk of all-cause death and cardiovascular death (CV death). The main safety outcomes were composite of serious adverse renal outcomes and volume depletion.

We performed the meta-analyses outcomes using Review Manager 5.3 software (Cochrane Network) and computed the pooled risk ratios (RR) or mean difference (MD) with corresponding 95% confidence intervals (CI) were synthesized. Between-study heterogeneity was tested using the chi-square-based Q test and I^2^ statistics. If I^2^ > 50%, it was considered as significant heterogeneity, in which case we chose the random-effects model to conduct the pooled analysis. If I^2^ was ≤50%, it showed no severe heterogeneity, so the fixed-effects model was used. We did not assess for publication bias using a funnel plot because of an insufficient number of studies.

## Results

The differences in characteristics between the patients taking and not taking sacubitril/valsartan at baseline were shown in Table 1. No significant differences were observed in age, sex, NYHA functional class, the use of mineralocorticoid receptor antagonists, and beta-blockers between the 2 groups. Compared to those without ARNI treatment, patients treated with ARNI had significantly lower ejection fraction, systolic blood pressures, heart rate, lower levels of NT-proBNP, and worse kidney function. Such patients were more common with higher body mass index and have been linked to a greater likelihood of receiving implantable cardioverter-defibrillator or cardiac resynchronization therapy (Table 1).

**Table 1:** Baseline characteristics in patients taking and not taking a neprilysin inhibitor.

	**Patients not taking a neprilysin inhibitor (*n* = 7239)**	**Patients taking a neprilysin inhibitor (*n* = 1235)**	***P-*Value**
Age (year)	66.65 ± 10.94	66.26 ± 11.23	0.2488
Women—*n* (%)	1727 (23.86)	275 (22.27)	0.232
Race—*n* (%)			0.000
White	5063 (69.94)	899 (72.79)	
Black	378 (5.22)	105 (8.50)	
Asian	1613 (22.28)	175 (14.17)	
Other	185 (2.56)	56 (4.53)	
Region—n (%)			0.000
North America	722 (9.97)	380 (30.77)	
Latin America	1870 (25.83)	233 (18.87)	
Europe	3054 (42.19)	453 (36.68)	
Asia	1465 (20.24)	124 (10.04)	
Other	128 (1.77)	45 (3.64)	
NYHA functional class-n (%)			0.498
II	5118 (70.70)	885 (71.66)	
III/IV	2121 (29.3)	350 (28.34)	
Body mass index (kg/m^2^)	27.92 ± 5.70	28.98 ± 6.02	<0.0001
LV ejection fraction (%)	29.87 ± 6.67	27.40 ± 6.67	<0.0001
Systolic blood pressure (mmHg)	122.77 ± 16.04	116.748 ± 15.22	<0.0001
Heart rate (beats/min)	71.7 ± 11.78	69.71 ± 11.348	<0.0001
NT-proBNP (pg/mL)	1663.59 ± 1576.18	1,511.50 ± 1354.27	0.0014
eGFR (mL/min/1.73 m^2^)	64.38 ± 20.53	62.746 ± 20.33	0.0096
Treatment of heart failure			
Cardiac glycosides	1290 (17.82)	191 (15.47)	0.047
Mineralocorticoid receptor antagonist	5152 (71.17)	879 (71.17)	1.000
Beta-blocker	6924 (95.65)	1167 (94.49)	0.075
Implantable cardioverter-defibrillator^a^	1576 (21.77)	548 (44.37)	0.000
Cardiac resynchronization therapy^b^	593 (8.19)	326 (26.40)	0.000

*Values are mean SD, n (%). NYHA, New York Heart Association; LV, left ventricular; NT-proBNP, N-terminal prohormone B-type natriuretic peptide; eGFR, estimated glomerular filtration rate*.

a *Implantable cardioverter defibrillator (ICD) or cardiac resynchronization therapy-defibrillator (CRT-D)*.

b *Cardiac resynchronization therapy with or without a defibrillator*.

The efficacy and safety of the combination therapy of SGLT-2 inhibitors and ARNI in patients with HFrEF were explored using meta-analysis. The statistical analysis included 1,235 patients treated with ARNI and 7,239 patients who were not. Based on the published dataset, we then categorized all these patients into 4 groups: combined treatment with SGLT-2 inhibitors and ARNI (590), SGLT-2 inhibitors monotherapy (3646), ARNI monotherapy (645), and placebo (3593) and conducted a four-arm study to compare the efficacy and safety of the combination therapy vs. single agent.

**Figure 2:**
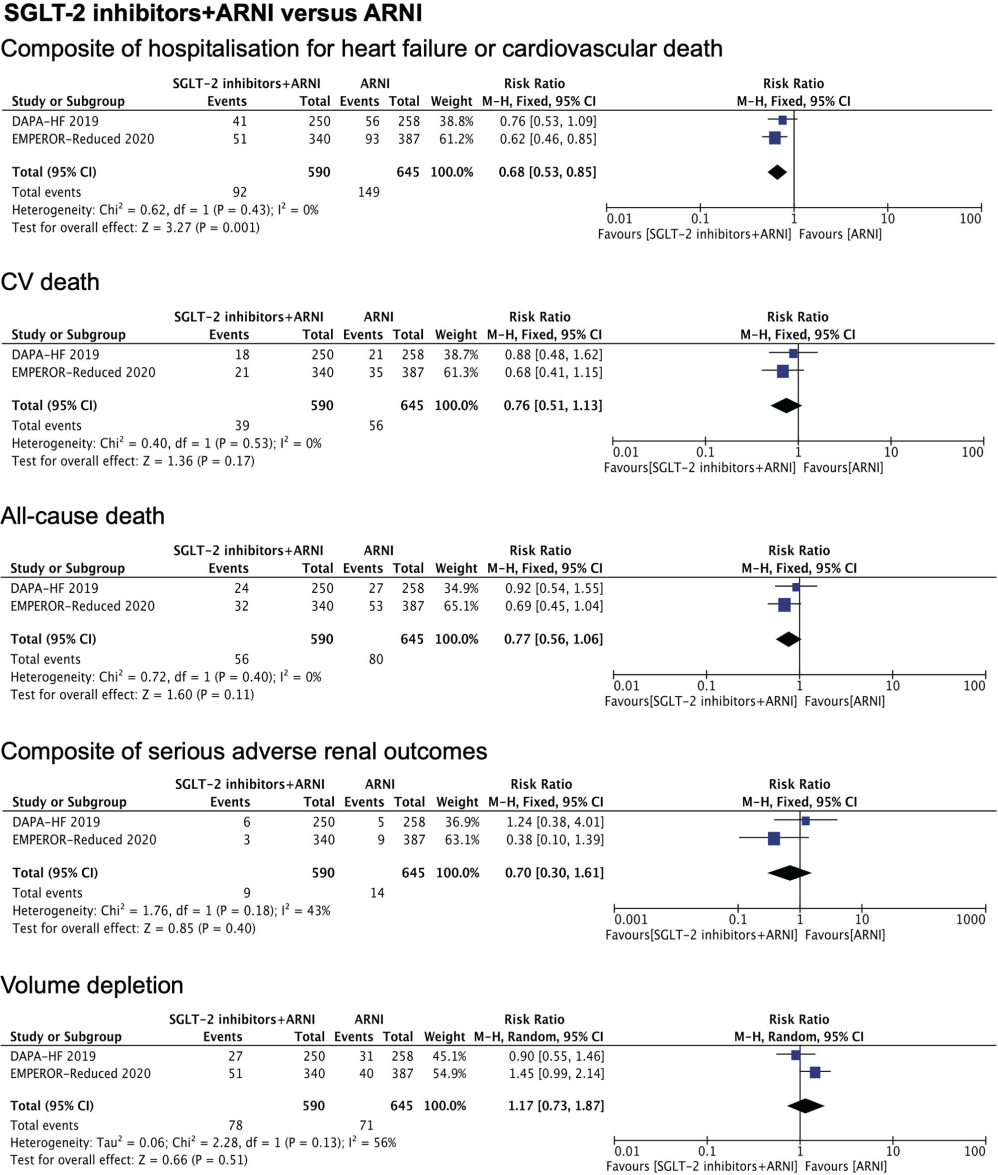
Meta-analysis of DAPA-HF and EMPEROR-Reduced trials. Figure 2 shows the therapeutic effects of combination therapy compared with ARNI monotherapy on a composite of hospitalization for heart failure or cardiovascular death, CV death, all-cause death, composite of serious adverse renal outcomes, and volume depletion. The composite of serious adverse renal outcome was defined as chronic dialysis or renal transplantation or a sustained reduction (≥40%) in the eGFR or a sustained eGFR of <15 ml/min/1.73 m^2^ in patients with a baseline eGFR of ≥30 ml/min/1.73 m^2^ or a sustained eGFR of <10 ml/min/1.73 m^2^ in those with a baseline eGFR of <30 ml/min/1.73 m^2^ in EMPEROR-Reduced trial. For the DAPA-HF trail, it was defined as a continuing decline of the eGFR (≥50%) for at least 28 days, end stage renal disease (ESRD), or death from renal causes. ESRD was defined as an eGFR of <15ml/min/1.73 m^2^ that was sustained for at least 28 days, sustained (≥28 days) dialysis treatment, or renal transplantation. eGFR, estimated glomerular filtration rate; RR, rate ratio.

**Figure 3:**
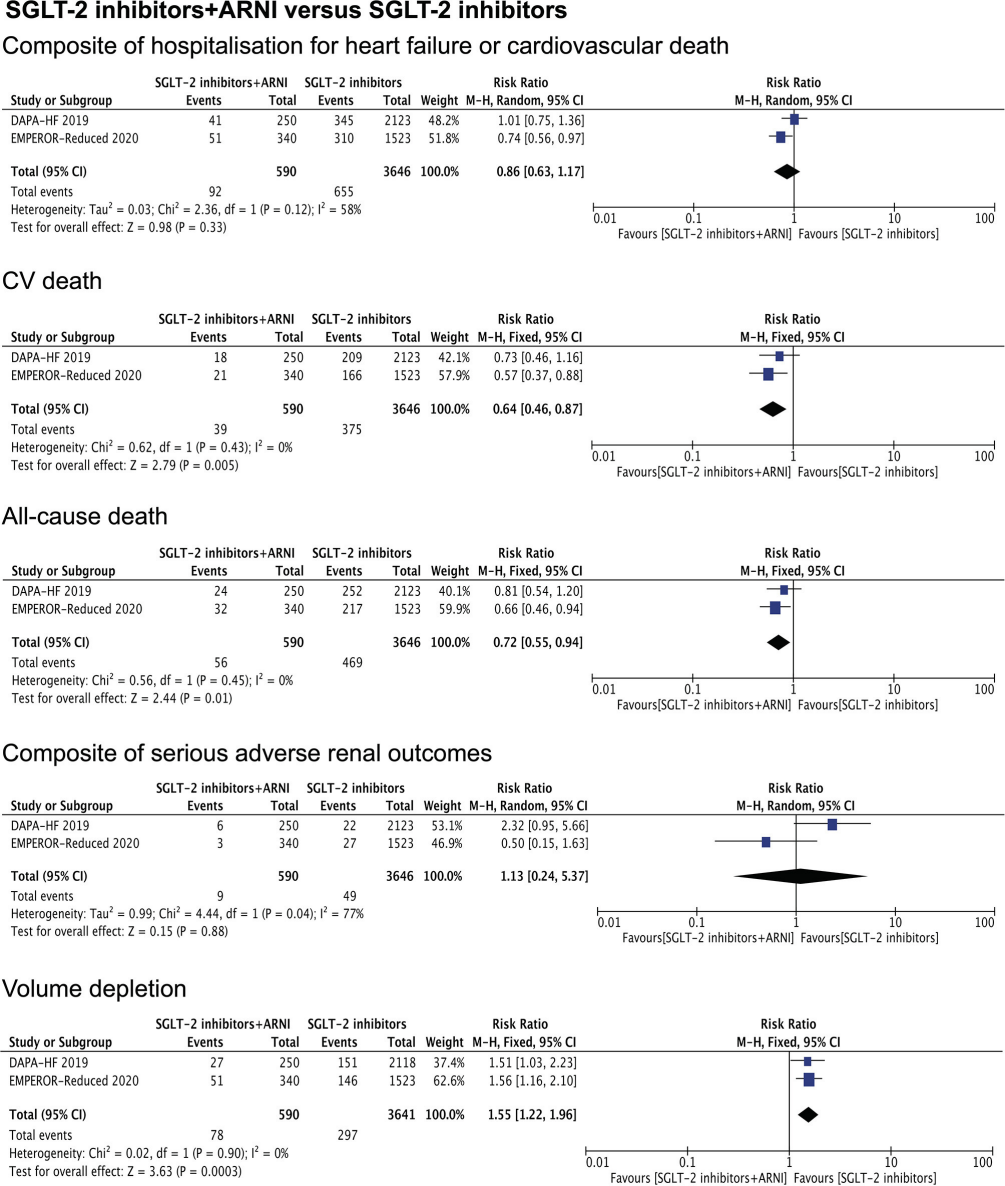
Shows the therapeutic effects of combination therapy compared with SGLT-2 inhibitors monotherapy on a composite of hospitalization for heart failure or cardiovascular death, CV death, all-cause death, composite of serious adverse renal outcomes, and volume depletion.

Our meta-analysis results showed a significant difference between the combination therapy vs. ARNI monotherapy in the rate of hospitalization for heart failure or cardiovascular death (RR.68, 95% CI.53 to.85, *P* = 0.001). There was no difference in the benefit of the combination therapy with respect to CV death, all-cause death, and volume depletion, compared with ARNI monotherapy (Figure 2). When compared with SGLT-2 inhibitors monotherapy, it was revealed that the combination therapy was superior in reducing the risk of CV death (RR.64, 95% CI.46 to.87, *P* = 0.005) and all-cause death (RR.72, 95% CI.55 to.94, *P* = 0.01). By contrast, patients treated with the combination therapy had a significantly greater rate of volume depletion compared to SGLT-2 inhibitors monotherapy (RR 1.55, 95% CI 1.22 to 1.96, *P* = 0.0003). No significant difference was found between the two groups in terms of a composite of hospitalization for heart failure or cardiovascular death (*P* = 0.33) (Figure 3).

## Discussion

The results of the pooled analysis demonstrated that the combined therapy with SGLT-2 inhibitors and ARNI significantly outperforms ARNI for reducing the rate of a composite of hospitalization for heart failure or cardiovascular death. The superiority of the combined therapy is also exhibited to SGLT-2 inhibitors for the reduction of CV death and all-cause death, however, at the cost of higher incidence of volume depletion. We also conducted the analysis of the combination therapy vs. monotherapy (any single drug). It is found that there is a significant reduction in CV death and all-cause death in the combination group (Supplemental Figure 1).

Previous studies have shown that sacubitril/valsartan increased levels of mature NPs and plasma cGMP, providing evidence that ARNI functions through the NP-cGMP-PKG axis (14–17), apart from the effects of renin-angiotensin system inhibition and diuresis. SGLT-2 inhibitors are a unique class of safe and effective hypoglycemic drugs that inhibit the reabsorption of filtered sodium and glucose in the proximal renal tubular, increasing urinary glucose, sodium, and water excretion, and reducing blood glucose level, body weight, and blood pressure, as well as protecting the renal function *via* renal tubular-glomerular feedback mechanism (18). The potential mechanisms of the effect of SGLT-2 inhibitors on heart failure have not yet been clearly elucidated (19–21). SGLT-2 inhibitors optimize myocardial energy metabolism and bioenergetics to inhibit sodium-hydrogen exchange and cardiac fibrosis and decrease the secretion of adipokine leptin (20, 22).

Given the apparent benefit of sacubitril/valsartan and SGLT-2 inhibitors for patients with HFrEF, both classes of drugs are recommended for these patients. But no previous studies had enrolled enough patients with a combined use of sacubitril/valsartan and SGLT-2 inhibitors to evaluate whether one of the two classes of drugs altered the effects of the other. The sub-analysis of the DAPA-HF and EMPEROR-Reduced trials presented inconsistent results in the aspect of the benefits of the combined therapy of SGLT-2 inhibitors and ARNI (10, 11). In addition, some similar mechanisms overlap in the effects of the two drugs on diuresis and glomerular perfusion. The safety issues are concerned with volume depletion and renal function, especially for patients with mild to moderate renal dysfunction.

Therefore, we conducted this meta-analysis to evaluate the effects of the combination treatment on the composite endpoints of hospitalization for heart failure and cardiovascular death, and CV death, all-cause death, and other relevant clinical endpoints in patients with HFrEF were analyzed as well.

The evidence from the study supports the current recommendation for the combined use of SGLT-2 inhibitors and ARNI for patients with HFrEF, since it could bring more benefits, compared with the ARNI monotherapy, in respect of composite of hospitalization for heart failure or cardiovascular death, not accompanied by severe adverse renal events and volume depletion. While compared with SGLT-2 inhibitors monotherapy, a significant reduction in the events of CV death and all-cause death was observed in the combined therapy. It is worthwhile to note that the patients who were treated with sacubitril/valsartan might be more severely ill at baseline. Compared with the patients without taking sacubitril/valsartan, those already taking the drug at baseline had lower ejection fraction, lower eGFR, and higher frequency of receiving implantable cardioverter-defibrillator or cardiac resynchronization therapy. The serious adverse renal events were not increased, whereas the incidence of volume depletion was higher in the combined therapy group. The results suggest that the volume and blood pressure should be closely monitored for the duration of the combined therapy with SGLT-2 inhibitors and ARNI for patients with HFrEF.

This meta-analysis has several limitations. The results of the study were limited by the inclusion of only two trials and some of the outcomes were heterogeneous (I^2^ ≥ 50%). Subgroup analysis could not be performed due to the low number of studies included. In the future, larger, multicenter, and randomized studies are expected to present more evidence for the combination therapy of SGLT-2 inhibitors and ARNI in patients with HFrEF. Second, although the DAPA-HF and EMPEROR-Reduced trials were randomized, double-blind studies, but when we divided them into 4 groups: combined treatment with SGLT-2 inhibitors and ARNI (590), SGLT-2 inhibitors monotherapy (3646), ARNI monotherapy (645), and placebo (3593), there was a large difference in numbers between the four groups, the total database with the use of ARNI is much smaller than those not taking a neprilysin inhibitor. Third, though both DAPA-HF and EMPEROR-Reduced trials included patients with HFrEF, the baseline characteristics of patients in the EMPEROR-Reduced trials demonstrated that worse cardiac dysfunction, higher levels of NT-proBNP, and poorer renal function, and the percentage of taking sacubitril/valsartan was much higher than in DAPA-HF. Meanwhile, the incidence of endpoint events in patients in the EMPEROR-Reduced trial was particularly higher than in DAPA-HF. This is perhaps a possible reason that contributes to heterogeneity. In addition, without access to obtain patient-level data and follow-up data, we had limited power to evaluate differences in all outcomes.

In conclusion, the effects of combined therapy with sacubitril/valsartan and SGLT-2 inhibitors were particularly well defined. Volume depletion is among the adverse reactions identified during the use of the two medications. But the sample size of concurrent trials is relatively small, additional evidence is needed to strengthen the existing conclusions.

## Data Availability Statement

The original contributions presented in the study are included in the article/Supplementary Material, further inquiries can be directed to the corresponding author.

## Author Contributions

All authors listed have made a substantial, direct, and intellectual contribution to the work and approved it for publication.

## Funding

This work was supported by the National Major Science and Technology Projects of China (2018YFC2000301).

## Conflict of Interest

The authors declare that the research was conducted in the absence of any commercial or financial relationships that could be construed as a potential conflict of interest.

## Publisher's Note

All claims expressed in this article are solely those of the authors and do not necessarily represent those of their affiliated organizations, or those of the publisher, the editors and the reviewers. Any product that may be evaluated in this article, or claim that may be made by its manufacturer, is not guaranteed or endorsed by the publisher.
