# Supplementary figures and images for: The Efficacy and Safety of the Combined Therapy of Sodium-Glucose Co-Transporter-2 Inhibitors and Angiotensin Receptor-Neprilysin Inhibitor in Patients With Heart Failure With Reduced Ejection Fraction: A Meta-Analysis of the EMPEROR-Reduced and DAPA-HF Sub-Analysis
Source: Front Cardiovasc Med. 2022 May 18;9:882089. doi: 10.3389/fcvm.2022.882089 (PMC9157547; doi:10.3389/fcvm.2022.882089)

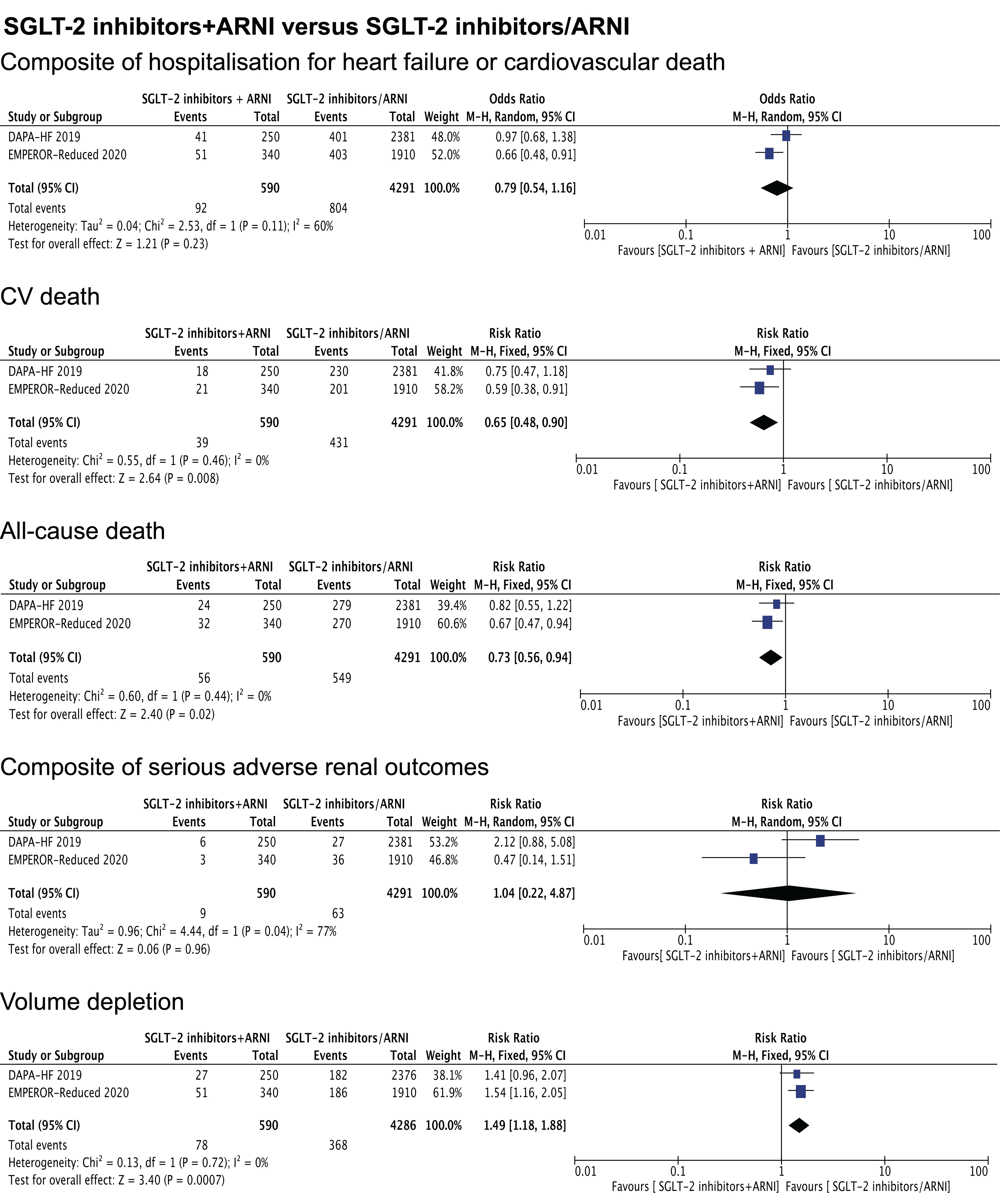

Supplement: Supplementary file 1 [file Image_1.jpg]
